# Quercetin Modulates IGF-I and IGF-II Levels After Eccentric Exercise-Induced Muscle-Damage: A Placebo-Controlled Study

**DOI:** 10.3389/fendo.2021.745959

**Published:** 2021-11-03

**Authors:** Paolo Sgrò, Roberta Ceci, Marco Lista, Federica Patrizio, Stefania Sabatini, Francesco Felici, Massimo Sacchetti, Ilenia Bazzucchi, Guglielmo Duranti, Luigi Di Luigi

**Affiliations:** ^1^Endocrinology Unit - Department of Movement, Human and Health Sciences, Università degli Studi di Roma “Foro Italico”, Roma, Italy; ^2^Laboratory of Biochemistry of Movement - Department of Movement, Human and Health Sciences, Università degli Studi di Roma “Foro Italico”, Roma, Italy; ^3^Laboratory of Exercise Physiology - Department of Movement, Human and Health Sciences, Università degli Studi di Roma “Foro Italico”, Roma, Italy

**Keywords:** anabolic factors, anti-inflammatory, antioxidant, skeletal muscle mass recovery, eccentric exercise

## Abstract

**Background:**

Prolonged or unaccustomed eccentric exercise may cause muscle damage and depending from its extent, this event negatively affects physical performance.

**Objectives:**

The aim of the present investigation was to evaluate, in humans, the effect of the flavonoid quercetin on circulating levels of the anabolic insulin-like growth factor 1 (IGF-I) and insulin-like growth factor 2 (IGF-II), produced during the recovery period after an eccentric-induced muscle damage (EIMD).

**Methods:**

A randomized, double-blind, crossover study has been performed; twelve young men ingested quercetin (1 g/day) or placebo for 14 days and then underwent an eccentric-induced muscle damaging protocol. Blood samples were collected, and cell damage markers [creatine kinase (CK), lactate dehydrogenase (LDH) and myoglobin (Mb)], the inflammatory responsive interleukin 6 (IL-6), IGF-I and IGF-II levels were evaluated before the exercise and at different recovery times from 24 hours to 7 days after EIMD.

**Results:**

We found that, in placebo treatment the increase in IGF-I (72 h) preceded IGF-II increase (7 d). After Q supplementation there was a more marked increase in IGF-I levels and notably, the IGF-II peak was found earlier, compared to placebo, at the same time of IGF-I (72 h). Quercetin significantly reduced plasma markers of cell damage [CK (p<0.005), LDH (p<0.001) and Mb (p<0.05)] and the interleukin 6 level [IL-6 (p<0.05)] during recovery period following EIMD compared to placebo.

**Conclusions:**

Our data are encouraging about the use of quercetin as dietary supplementation strategy to adopt in order to mitigate and promote a faster recovery after eccentric exercise as suggested by the increase in plasma levels of the anabolic factors IGF-I and IGF-II.

## 1 Introduction

Eccentric movements, characterized by the lengthening of skeletal muscle while producing force, are carried out in everyday life. Eccentric exercises, providing a potent stimulus to promote muscle growth and strength, are also an important part of training programs for athletes at both the professional and recreational level (1). Furthermore, programs that include eccentric contractions have gained a growing interest in several fields; indeed, for their positive effect on muscle mass, they are proposed to special populations such as aged individuals suffering sarcopenia or patients with neuromuscular disorders (2–5). However, the use of eccentric exercise has been often the object of contrasting views because of its potentially adverse effects; in fact, eccentric contractions, especially when unaccustomed, induce greater muscle damage than other types of exercise (6).

A wealth of work has investigated how muscle damage could be contained in order to take advantage of the benefits of eccentric exercise in improving muscle strength while limiting the extent of loss of function. To attenuate the exercise-induced muscle damage (EIMD), different nutritional and supplementation strategies have been attempted (6, 7). Nowadays the attention has shifted to nutraceuticals, bioactive compounds that it has been reported to play a significant role in maintaining muscle health. They act as potential therapeutic agents in a broad range of muscle atrophy models (8) and their long-term use seem safer respect to traditional therapeutic agents.

Among them, quercetin (Q), a plant derived flavonol from the flavonoids group of polyphenols has been considered a promising molecule in this topic (9). It has been known to exert a variety of bioactive effects that are intrinsically linked to its strong antioxidant and anti-inflammatory properties (10). However, its role in human studies is still debated due to the mixed results obtained (11–14).

To sum up the events following EIMD, it has been known that the injury initiates a regeneration response; satellite cells (a population of skeletal muscle stem cells) become activated, migrate to the site of damage, proliferate and then fuse with the injured fiber, in a process ultimately leading to muscle hypertrophy after repeated bouts of exercise (1, 15). In addition, other cells within the muscle altogether with the activity of immune cells, recruited in response to injury, contribute to muscle regeneration through the production of soluble factors, such as the insulin-like growth factors (IGFs) that are known to support satellite cell proliferation and differentiation (16). Investigating the role of IGFs in mediating some of the beneficial aspects of exercise especially with regard to post-exercise recovery and remodeling mechanisms for muscle tissue is a field of many ongoing research.

Particularly of interest are insulin-like growth factor (IGF)-I and IGF-II, both locally expressed from the muscle cell itself, as response to mechanical-stretch events. IGF-1 activates PI3K/Akt pathway and through the activation of rapamycin complex (mTOR) kinase stimulates protein synthesis and consequently muscle growth (17).

Furthermore, it has been reported that the overexpression of IGF-1 on the soleus muscle accelerates muscle regeneration (18). Regarding the role of IGF-II in muscle repair, so far is not fully understood; however, it is known that the expression of IGF-I and IGF-II occurs at different stages of muscle regeneration, with IGF-I expression preceding that of IGF-II (19).

The consumption of polyphenols is largely proposed as a therapeutic strategy to control muscle atrophy and/or to ameliorate muscle mass and strength *via* the attenuation of oxidative stress, inflammation, and the inhibition of muscle atrophy related genes and the activation of IGF-1 signaling pathway (8, 9).

While it is known that the Q supplementation may help to mitigate early symptoms of EIMD, conversely, whether Q affects the following adaptive remodeling process by modulating IGF-I and IGF-II levels has not been investigated yet.

The aim of this study was to determine whether Q supplementation, during the recovery phase, influences the levels of anabolic hormones IGF-I and IGF-II and mitigates muscle damage.

For this purpose, we conducted, on healthy young men, a randomized, double-blind, and placebo-controlled intervention study using Q (1 g/day) and placebo (PLA) for 14 days. After that, the subjects underwent to an eccentric exercise protocol able to induce muscle damage.

So, taking into account that EIMD develops its clinical manifestation beyond 24 hours post-exercise, we conducted the present study at different time points: from 24 hours to 7 days. Blood samples from placebo/Q treatments were collected before/after supplementation and post EIMD, to evaluate: 1) cellular damage markers [creatine kinase (CK), lactate dehydrogenase (LDH), myoglobin (Mb)]; 2) inflammatory status [interleukin 6 (IL6)]; and 3) the anabolic IGF-I and IGF-II plasma levels (20–24).

## 2 Materials and Methods

### 2.1 Subjects

Twelve healthy moderately active young men were recruited among students of our University with an age range between 18-30 and with a healthy weight BMI (**Table 1**). The exclusion criteria included: a) history or signs of metabolic, renal, cardiac or neurological diseases; b) usage of any dietary supplement for at least 6 months prior to the study; i.e. amino acids, vitamins and/or antioxidants in particular with quercetin containing foods; c) usage of medications, anabolic agents; d) the presence of major stress events, contraindicating and/or interfering with the experimental procedures and evaluations. No exercise, or major stress events were allowed starting at 48 hours before each test.

**Table 1:** Subjects characteristics.

Subjects characteristics (n = 12)	
**Age (years)**	25.67 ± 3.87
**Height (m)**	1.78 ± 0.07
**Weight (Kg)**	77.42 ± 5.11
**BMI (kg∙ m^-2^)**	24.56 ± 1.30

BMI, body mass index.

Participants were asked to continue their habitual exercise and nutritional routines. In addition, to avoid the ingestion of foods containing Q and other antioxidant properties, from 1 week before and during the study period, nutritional supplements and ergogenic aids were not allowed. Subjects were also asked to refrain from taking anti-inflammatory medications while taking part in the study.

Subjects were fully informed of the experimental protocol as well as the possible risks and discomforts of the investigation. All subjects gave their informed consent for inclusion before they participated in the study. The study was conducted in accordance with the Declaration of Helsinki, and the protocol was approved by the Ethics Committee of the University of Rome “Sapienza” approved the protocol of the study under the process number 2621/15.

### 2.2 Experimental Overview and Sample Collection

This study used a double-blind, placebo-controlled, crossover design. An equal number of volunteers were randomized to quercetin (Q) or placebo (PLA) treatment and then crossed over to the opposite condition after a 3-week washout period. On the first visit, volunteers were familiarized with the experimental procedures. The arm exposed to the eccentric protocol [right (R) or left (L)] was reversed when the protocol was repeated with the other treatment. The association between treatment (Q or PLA) and arm (R or L) was also randomly assigned so that the limb dominance was not uniquely associated with the supplementation.

Volunteers were randomly assigned to Q or PLA. Starting from the following day, for 14 consecutive days, participants ingested 2 capsules containing 500 mg of quercetin aglycone in crystalline powder (Farmalabor Srl, Milan, Italy), one at breakfast and one 12 hours later, to achieve a daily experimental dose of 1 g according to bioavailability testing already reported in humans (25). Placebo capsules (Farmalabor Srl, Milan, Italy) were indistinguishable by taste and appearance from those of Q. Volunteer compliance was monitored daily by the investigators.

After 2 weeks Q/PLA supplementation, participants underwent eccentric exercise protocol. Each participant completed 10 bouts (separated by a 30 s-rest) of 10 maximal lengthening contractions of the elbow flexors. Each eccentric contraction lasted 2 s followed by a 6-s rest in which the dynamometer arm returned automatically backward (26). The dynamometer was set to an angular velocity of 45°/s and participants were instructed to resist maximally during the whole ROM (100° starting from 40° to 140°). In order to assess the muscle strength before, after the eccentric protocol and during the recovery period, the maximal voluntary isometric contraction (MVC) of the elbow flexors was calculated as already described (26). Briefly, the MVC task consisted of rapidly increasing the force to a maximum and to maintain it for at least 2-3 s before relaxing. A minimum of three maximal attempts were performed separated by 5 min to recover from fatigue.

**Figure 1:**
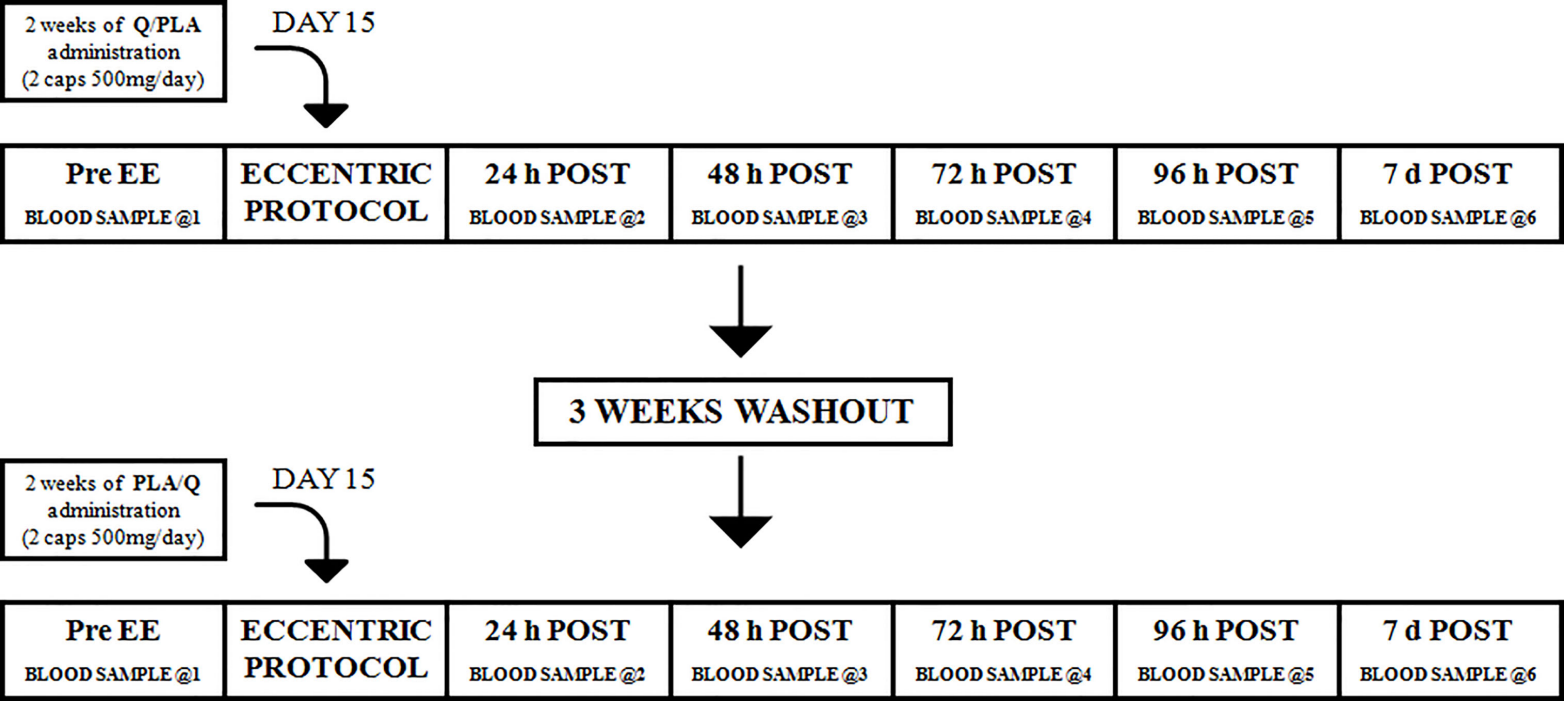
Flow diagram of the study. Twelve subjects were randomly assigned to quercetin group (n = 6) or to placebo group (n = 6) and then crossed over to the opposite condition. Subjects were given two caps of quercetin (1 g/d) or placebo for 2 weeks. On day 15, subjects were submitted to eccentric exercise (EE). Blood collections were performed before the EE (Pre EE), 24, 48, 72 and 96 hours (24 h – 96 h), and 7 days after EE (7 d).

Different parameters were assessed after 14 days of Q/PLA intake (Pre EE) and at different recovery times after the eccentric protocol (24, 48, 72, 96 hours and 7 days after EE) (**Figure 1**).

At each experimental point, blood samples (10 mL for each draw) were collected, and plasma was immediately separated (3 000 rpm×10 minutes, +4°C) and stored at –80°C until hormonal assays were performed (27).

### 2.3 Biochemical Analyses

All chemical reagents were purchased from Sigma-Aldrich Chemical (St. Louis, MO, USA).

Plasma creatine kinase (CK) and lactate dehydrogenase (LDH) activity was determined spectrophotometrically, according to manufactory recommendations, by a manual procedure using a commercial test kit (Greiner Diagnostic GmbH, Bahlingen, Germany) as previously described (28).

Plasma myoglobin (Mb) levels were determined spectrophotometrically, according to manufactory recommendations, by a manual procedure using a commercial test kit (ThermoFisher-Invitrogen, Waltham, MA, USA).

Plasma interleukin-6 (IL-6) concentration was determined using a quantitative sandwich enzyme immunoassay technique, according to the manufacturer’s instructions (R&D Systems Europe, Abingdon, UK) as previously described (20). IL-6 concentration (pg/mL) was calculated by extrapolation from a standard curve generated with serial concentrations of the standard protein solution provided by the manufacturer; values below the standard range were discarded.

### 2.4 Insulin-Like Growth Factor 1 and Insulin-Like Growth Factor 2 Plasma Levels

Plasma levels of IGF-I and IGF-II were determined using the Bio-Plex Suspension Array System (Bio-Rad Laboratories Hercules, California USA) (29). The intra-assay and inter-assay coefficients of variation of the hormones evaluated were 2% and 6% for IGF-I and 2% and 4% for IGF-II, respectively. The sensitivity of the method was 0.10 ng/mL for IGF-I, and 0.13 ng/mL for IGF-II. The lower limit of assay quantification is 0.25 ng/mL for IGF-I and 0.19 ng/mL for IGF-II while the upper limit for assay quantification is 87 and 35 ng/mL respectively. Each sample was tested in triplicate.

### 2.5 Statistical Analysis

For CK, LDH, Mb, IL-6, IGF-I and IGF-II six separate factorial ANOVA with repeated measure with treatment (e.g., placebo, quercetin) and time (e.g., Pre EE, 24, 48, 72, 96 hours and 7 days) as factors were performed. When significant interaction (i.e., treatment for time) was observed, follow-up tests were conducted running separate repeated measures ANOVA for treatment to explore the different effects of time in the two-treatment condition. *Post-hoc* comparisons were performed by means of Fisher’s LSD test and the Bonferroni alpha level correction was applied. Statistical analyzes were performed on SPSS (IBM SPSS, Armonk, NY, USA). The significance level for all statistical tests was set at p <0.05.

## 3 Results

### 3.1 Muscle Strength

As expected, the maximum strength of the arm flexor muscles was decreased after the eccentric protocol in both conditions. However, this decrease was less marked in Q condition (-43.4% +/- 17.6 in Q compared to -51.2% +/- 16.2 in PLA) and MVC recovery was greater in all recovery time points (24h: -25.4% +/- 14.3 Q -35.8% +/- 13.8 PLA; 48h: -24.5% +/- 14.1 Q -33.6% +/- 12.6 PLA; 72h: -17.8% +/- 12.5 Q -29.8% +/- 13.7 PLA; 96h: -16.3%+/- 11.9 Q -27.1% +/- 12.1 PLA; 7d: -10.5% +/- 9.8 Q -18.7% +/- 11.7 PLA).

### 3.2 Quercetin Supplementation Decreases Markers of Muscle Damage After EE

#### 3.2.1 Creatine Kinase Levels

Quercetin supplementation showed differences in CK levels with regard to: the treatment effect (p <0.005), the effect of time (p <0.0001), and the time effect * treatment (p <0.001).

The interaction between pre-exercise (Pre EE) and the different recovery times, in the Placebo group showed differences at 48 h - 7 d post eccentric protocol (p <0.01).

The quercetin group showed differences with respect to the Pre EE at 48 h - 7 d post eccentric protocol (p <0.01).

**Figure 2:**
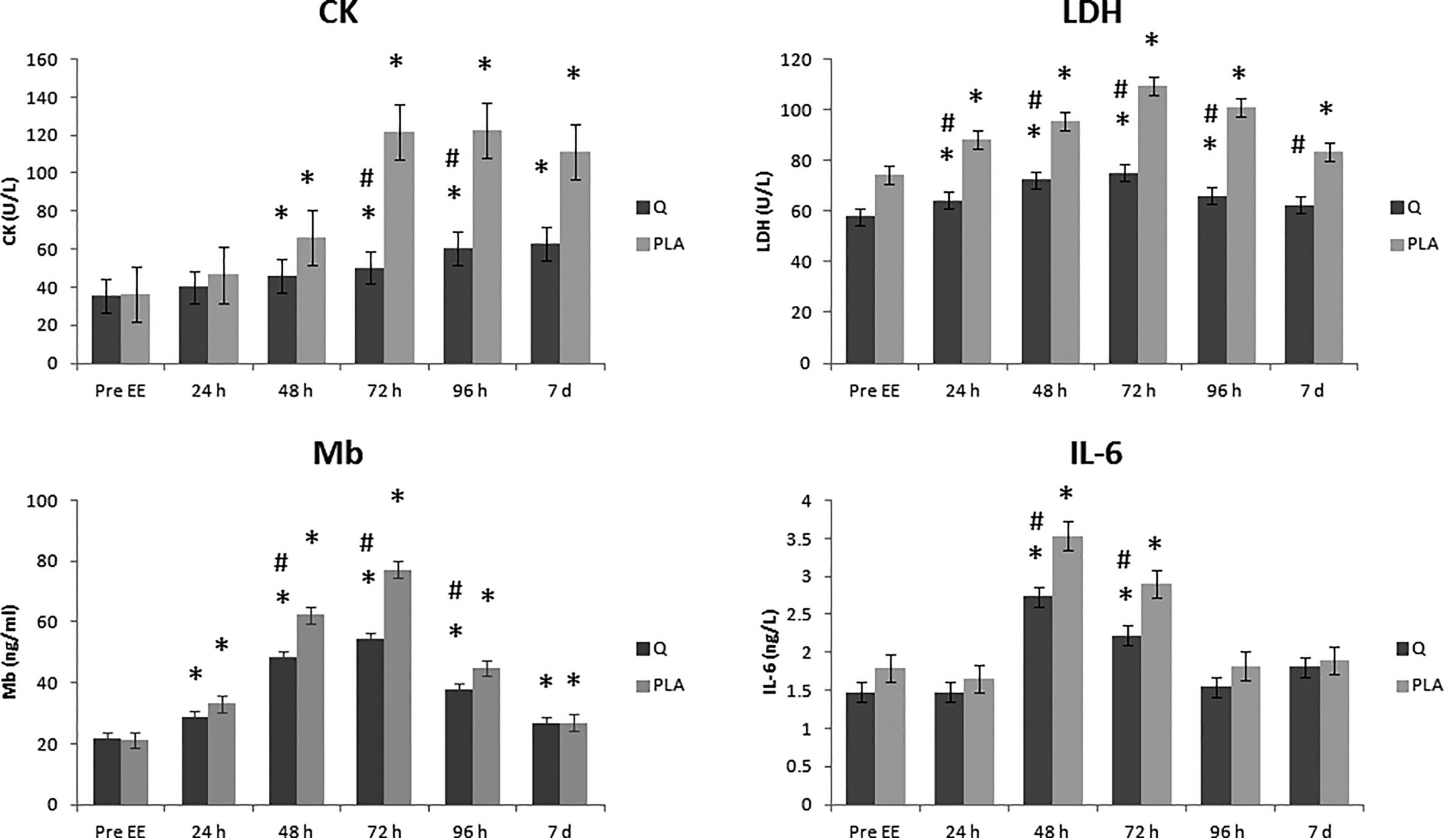
Effect of quercetin supplementation on creatine kinase (CK), lactate dehydrogenase (LDH), myoglobin (Mb) and Interleukin-6 (IL-6) plasma levels. The levels of creatine kinase (CK), lactate dehydrogenase (LDH), myoglobin (Mb) and Interleukin-6 (IL-6), were measured in plasma of quercetin/placebo supplemented subjects before the eccentric exercise (Pre EE) and after 24, 48, 72 and 96 hours (24 h – 96 h) and after 7 days (7 d) recovery time. Values are expressed as means ± pooled SEM. n =12 for each group. *P < 0.05 *vs* Pre EE and ^#^P < 0.05 *vs* placebo.

The interaction between the two groups (quercetin *vs* placebo) at different recovery times showed differences after 72 h (p <0.05; 59% lower level in quercetin group) and 96 h post eccentric protocol (p <0.01; 49% lower level in quercetin group) (**Figure 2**).

#### 3.2.2 Lactate Dehydrogenase Levels

Quercetin supplementation showed differences in LDH levels with regard to: the treatment effect (p <0.001), the effect of time (p <0.001), and the time effect * treatment (p <0.001).

The interaction between pre-exercise (Pre EE) and the different recovery times, in the placebo group, the interaction between Pre EE and the different recovery times, showed differences at all the time post eccentric protocol (p <0.05).

The quercetin group showed a statistically significant difference with respect to the Pre EE at 24 – 96 h post eccentric protocol (p <0.005). After 7 days post EE no statistically significant levels of LDH were found.

The interaction between the two groups (quercetin *vs* placebo) at different recovery times showed differences between the Quercetin group and the Placebo group at all the time post eccentric protocol (p<0.005) with the maximal difference at 96 h (p<0.0001; 39% lower level in quercetin group) (**Figure 2**).

#### 3.2.3 Myoglobin Levels

Quercetin supplementation showed differences in Mb levels with regard to: the treatment effect (p <0.05), the effect of time (p <0.01), and the time effect * treatment (p <0.05).

The interaction between pre-exercise (Pre EE) and the different recovery times, in the Placebo group showed differences at 24 h - 7 d post eccentric protocol (p <0.01).

The quercetin group showed differences with respect to the Pre EE at 24 h - 7 d post eccentric protocol (p <0.01).

The interaction between the two groups (quercetin *vs* placebo) at different recovery times showed differences after 48 h (p <0.01; 30% lower level in quercetin group) and 72 h post eccentric protocol (p <0.01; 23% lower level in quercetin group) (**Figure 2**).

#### 3.2.4 Interleukin-6 Levels

Quercetin supplementation showed differences in IL-6 levels with regard to: the treatment effect (p <0.005), the effect of time (p <0.0001), and the time effect * treatment (p <0.05).

The interaction between the Pre EE and the different recovery times, in the placebo group showed differences with respect to the 48h and 72 h post eccentric protocol time (p <0.001; 97% and 61% increase respectively).

The quercetin group showed differences with respect to the Pre EE at the 48 h and 72 h post eccentric protocol time (p <0.001; 85% and 50% increase respectively) (**Figure 2**).

The interaction between the two groups (quercetin *vs* placebo) at different times showed differences between the Quercetin group and the Placebo group at the time of 48 h (p <0.05; 22% lower level in quercetin group) and 72 h post eccentric protocol (p <0.05; 23% lower level in quercetin group) (**Figure 2**).

### 3.3 Quercetin Supplementation Increases the Levels of Circulating Insulin-Like Growth Factors After EE

#### 3.3.1 Insulin-Like Growth Factor 1

Quercetin supplementation showed differences in IGF-I levels with regard to: the treatment effect (p <0.001), the effect of time (p <0.001), and the interaction time * treatment (p <0.001).

*Post-hoc* between pre-exercise (Pre EE) and the different recovery times, in the Placebo treatment showed differences respect to the 72 h – 7 d post eccentric protocol time (p <0.001). The quercetin treatment showed differences compared to Pre EE at 48 h – 7 d post eccentric protocol time (p <0.05).

**Figure 3:**
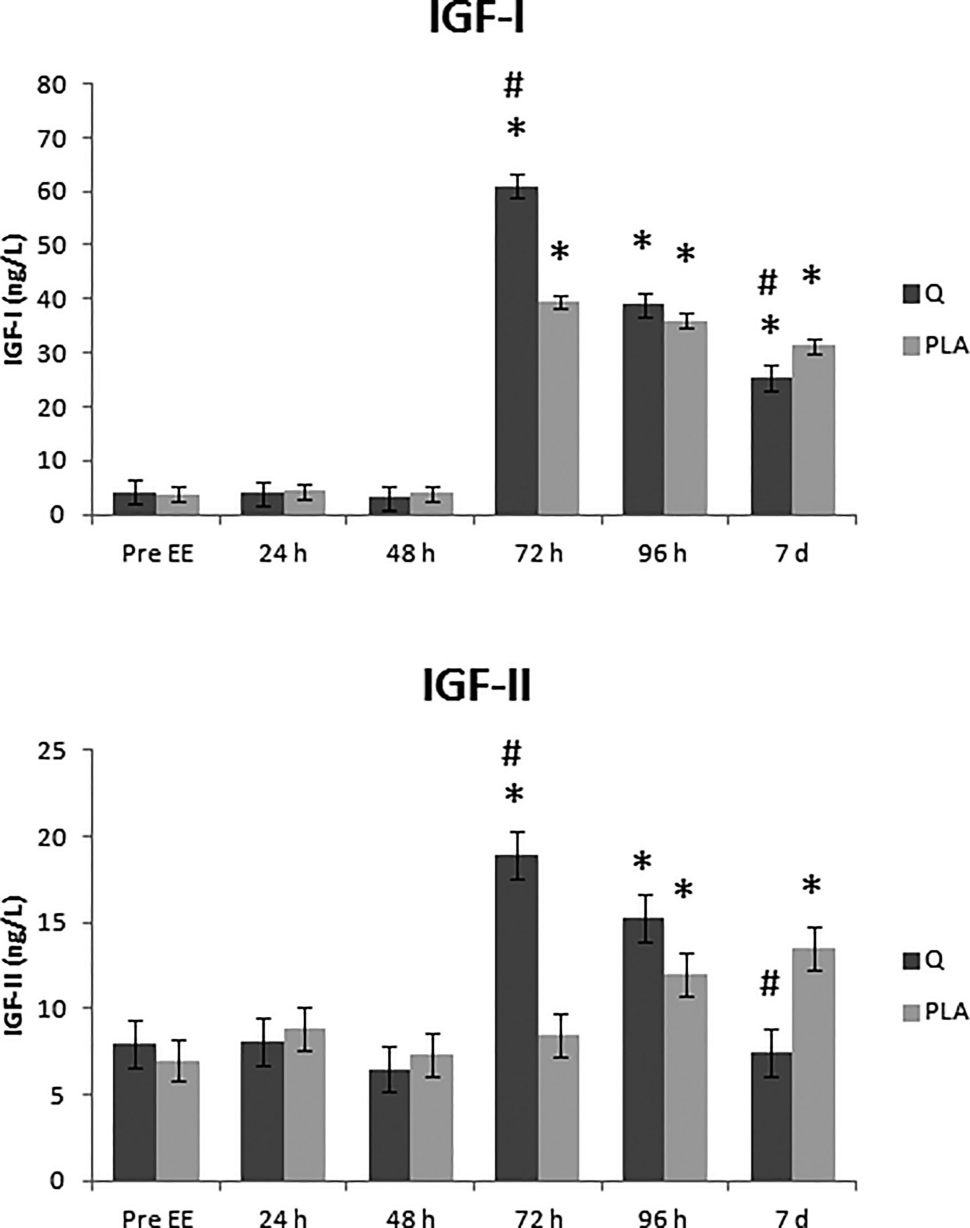
Effect of quercetin supplementation on insulin-like growth factor 1 (IGF-I) and insulin-like growth factor 2 (IGF-II) plasma levels. The levels of insulin-like growth factor 1 (IGF-I) and insulin-like growth factor 2 (IGF-II) were measured in plasma of quercetin/placebo supplemented subjects before the eccentric exercise (Pre EE) and after 24, 48, 72 and 96 hours (24 h – 96 h) and after 7 days (7 d) recovery time. Values are expressed as means ± pooled SEM. n =12 for each group. *P < 0.05 *vs* Pre EE and ^#^P < 0.05 *vs* placebo.

The comparison between the two treatments (quercetin *vs* placebo) showed differences after 72 h (p<0.005; 54% higher level in quercetin treatment) and 7 days post eccentric protocol (p<0.05; 19% lower level in quercetin treatment; **Figure 3**).

#### 3.3.2 Insulin-Like Growth Factor 2

Quercetin supplementation showed differences in IGF-II levels with regard to: the treatment effect (p <0.005), the effect of time (p <0.001), and the interaction time * treatment (p <0.001).

The comparison between pre-exercise (Pre EE) and the different recovery times, in the placebo treatment showed differences respect to the 96 h – 7 d post eccentric protocol time (p <0.05; **Figure 3**).

The quercetin treatment showed differences compared to Pre EE at 72 h and 96 h post eccentric protocol time (p <0.005). After 7 days post EE no statistically significant levels of IGF-II were found.

*Post-hoc* between the two treatments (quercetin *vs* placebo) at different recovery times showed differences after 72 h (p <0.005; 124% higher level in quercetin treatment) and 7 days post eccentric protocol (p <0.05; 45% lower level in quercetin treatment; **Figure 3**).

## 4 Discussion

The aim of the present investigation was to evaluate the effect of 14 days of supplementation with 1 g/d of quercetin on IGFs levels during the recovery period following an acute eccentric exercise.

As expected, we observed that an acute bout of eccentric exercise produced, in the recovery time post-exercise, sustained increases in the clinical markers of muscle damage, along with an increased level of the inflammatory responsive cytokine IL-6.

The results of the present study show that the use of chronic quercetin supplementation reduces biochemical markers of muscle damage and IL-6 levels during the recovery period compared to controls, while promoting an increase in IGFs levels therefore leading to faster recovery of muscle strength.

The regeneration process, which starts after the damage, includes the release of growth factors, such as the IGFs, hormones playing major roles in muscle growth and differentiation. A greater increase in their plasma levels was observed in our experimental condition, after EIMD, compared to placebo. As reported in literature, in placebo treatment the increase in IGF-I (72 h) preceded IGF-II increase (7 d). After Q supplementation a more marked increase in IGF-I and IGF-II levels was observed. Interestingly, the IGF-II peak was found earlier compared to placebo, concomitant with that of IGF-I (72 h).

It must be noted that the presence of quercetin accelerates the increases of IGF-II muscle damage-related. The explanation of this phenomenon is very complex given that to date it is not entirely clear what is the real function of IGF-II at muscular level. We can hypothesize that having effects similar to those of IGF-I and being necessary for the biological activity of myoblast determination protein 1 (MyoD), one of the earliest markers of myogenic commitment, also IGF-II is involved in muscle damage repair. In fact, MyoD transcription factor, requires the expression of IGF-II for fibroblast differentiation in myoblasts (30). In an *in-vitro* study conducted on rat soleus was observed that IGF-II, in addition to IGF-I, is expressed in myofibrils following eccentric load induced myofibrillar interruption (31).

Moreover, it has been demonstrated that the production of IGFs is specific for the type of training. IGF-I has positive effects on repair and muscle recovery after resistance training, stimulating local protein synthesis and preventing apoptosis. This anabolic effect seems to be increased in response to the mechanical stress induced by training (32, 33).

Even if the levels of circulating IGF-II are little investigated, a number of studies show that in adult life and in response to exercise its increase seems to be associated with endurance exercise (34, 35).

From what has been observed in our study, quercetin not only has antioxidant effects as already observed in the literature (36–45), but it would seem to have a conditioning effect also on the regulation of the secretion of similar insulin growth factors, which at local level play a fundamental role in hypertrophy, post-workout remodeling and post-workout muscle damage repair. Since these functions are directly related to the gain of muscular strength and the administration of quercetin not only has ergogenic effects but also reduces the loss of muscle strength associated with damage, this could be used to prevent damage and promote recovery.

It seems obvious that our final observations cannot be generalized since the subjects analyzed were moderately trained, and therefore we cannot know if this flavonoid induces the same effects in very trained or elite athletes.

As a limitation of the present study is the laboratory assay of total IGF-I instead of the analysis of the different IGF-I isoforms. In fact it is known that there are three different isoforms of IGF-I: two are produced by the muscle (IGF-IEa, IGF-IEc [MGF]) (46) and one by the liver (IGF-IEb) under direct control of the growth hormone (GH) (46). IGF-IEa, is similar in molecular structure to the circulating IGF-I secreted by the liver. The second isoform, called mechanical growth factor (MGF) or IGF-1Ec, is secreted in response to muscle contractions and muscle stretch. It performs its functions locally, stimulating proliferation, maturation and growth of muscle cells and of quiescent satellite cells (stem cells), after muscle damage (47).

Then, we were not able to assess what is the real contribution of the muscle in the production of IGFs but due to no significant changes in GH levels (data not shown) we can state that the increase of IGF-I and IGF-II is not GH-related accounting for a production and/or secretion by skeletal muscle itself of these two growth factors.

Moreover, it was not possible to obtain other biological samples such as muscle biopsies from the subjects. For this reason, it was not possible to evaluate the presence of fibrotic events or fibroblasts proliferation.

## 5 Conclusions

In conclusion, dietary supplementation with quercetin, possibly through its anti-inflammatory and antioxidant properties could be a way to prevent, mitigate and promote a faster recovery of EIMD by eccentric training. Our findings are specific to a sample of young, healthy, trained men. Considering that IGFs production are controlled by ROS, further studies are warranted to determine if similar or more pronounced results can be observed in different experimental conditions (e.g., age, gender, fitness status, type of stressor) and with different quercetin supplementation also in combination with other antioxidant and ergogenic substances.

## Data Availability Statement

The raw data supporting the conclusions of this article will be made available by the authors, without undue reservation.

## Ethics Statement

The studies involving human participants were reviewed and approved by Ethics Committee of the University of Rome “Sapienza”. The patients/participants provided their written informed consent to participate in this study.

## Author Contributions

PS: participated in the design of the study, contributed to data collection, data analysis and interpretation of results. RC: participated in the design of the study, contributed to data collection, data analysis and interpretation of results. ML: contributed to data collection and data analysis. FP: contributed to data collection and data analysis. SS: participated in the design of the study and contributed to interpretation of results. FF: participated in the design of the study and contributed to interpretation of results. MS: participated in the design of the study and contributed to interpretation of results. IB: participated in the design of the study, contributed to data collection and data analysis. GD: participated in the design of the study, contributed to data collection and data analysis and contributed to interpretation of results. LL: participated in the design of the study and contributed to interpretation of results. All authors contributed to the manuscript writing. All authors read and approved the final version of the manuscript. All authors were involved in the study design and revised the final version of the manuscript, with intervention in the analysis of data, statistical evaluation, and final interpretation of the results of this study. All authors contributed to the article and approved the submitted version.

## Conflict of Interest

The authors declare that the research was conducted in the absence of any commercial or financial relationships that could be construed as a potential conflict of interest.

## Publisher’s Note

All claims expressed in this article are solely those of the authors and do not necessarily represent those of their affiliated organizations, or those of the publisher, the editors and the reviewers. Any product that may be evaluated in this article, or claim that may be made by its manufacturer, is not guaranteed or endorsed by the publisher.
